# Ancestral SARS-CoV-2-specific T cells cross-recognize the Omicron variant

**DOI:** 10.1038/s41591-022-01700-x

**Published:** 2022-01-14

**Authors:** Yu Gao, Curtis Cai, Alba Grifoni, Thomas R. Müller, Julia Niessl, Anna Olofsson, Marion Humbert, Lotta Hansson, Anders Österborg, Peter Bergman, Puran Chen, Annika Olsson, Johan K. Sandberg, Daniela Weiskopf, David A. Price, Hans-Gustaf Ljunggren, Annika C. Karlsson, Alessandro Sette, Soo Aleman, Marcus Buggert

**Affiliations:** 1 Department of Medicine Huddinge, Center for Infectious Medicine, Karolinska Institutet, Stockholm, Sweden; 2 Center for Infectious Disease and Vaccine Research, La Jolla Institute for Immunology, La Jolla, CA USA; 3 Division of Clinical Microbiology, Department of Laboratory Medicine, Karolinska Institutet, Stockholm, Sweden; 4 Department of Hematology, Karolinska University Hospital, Stockholm, Sweden; 5 Department of Oncology-Pathology, Karolinska Institutet, Stockholm, Sweden; 6 Department of Infectious Diseases, Karolinska University Hospital, Stockholm, Sweden; 7 Division of Infection and Immunity, Cardiff University School of Medicine, University Hospital of Wales, Cardiff, UK; 8 Systems Immunity Research Institute, Cardiff University School of Medicine, University Hospital of Wales, Cardiff, UK; 9 Division of Infectious Diseases and Global Public Health, Department of Medicine, University of California, San Diego, La Jolla, CA USA; 10 Infectious Diseases and Dermatology Unit, Department of Medicine Huddinge, Karolinska Institutet, Stockholm, Sweden

**Keywords:** Infectious diseases, Cellular immunity, RNA vaccines, T cells

## Abstract

The emergence of the severe acute respiratory syndrome coronavirus 2 (SARS-CoV-2) Omicron (B.1.1.529) variant of concern (VOC) has destabilized global efforts to control the impact of coronavirus disease 2019 (COVID-19). Recent data have suggested that B.1.1.529 can readily infect people with naturally acquired or vaccine-induced immunity, facilitated in some cases by viral escape from antibodies that neutralize ancestral SARS-CoV-2. However, severe disease appears to be relatively uncommon in such individuals, highlighting a potential role for other components of the adaptive immune system. We report here that SARS-CoV-2 spike-specific CD4^+^ and CD8^+^ T cells induced by prior infection or BNT162b2 vaccination provide extensive immune coverage against B.1.1.529. The median relative frequencies of SARS-CoV-2 spike-specific CD4^+^ T cells that cross-recognized B.1.1.529 in previously infected or BNT162b2-vaccinated individuals were 84% and 91%, respectively, and the corresponding median relative frequencies for SARS-CoV-2 spike-specific CD8^+^ T cells were 70% and 92%, respectively. Pairwise comparisons across groups further revealed that SARS-CoV-2 spike-reactive CD4^+^ and CD8^+^ T cells were functionally and phenotypically similar in response to the ancestral strain or B.1.1.529. Collectively, our data indicate that established SARS-CoV-2 spike-specific CD4^+^ and CD8^+^ T cell responses, especially after BNT162b2 vaccination, remain largely intact against B.1.1.529.

## Abstract (web summary)

Peripheral ancestral SARS-CoV-2 spike-specific CD4^+^ and CD8^+^ T cells induced by BNT162b2 vaccination cross-react to the Omicron variant at higher levels than those induced by prior SARS-CoV-2 infection.

## Main

Natural infection with SARS-CoV-2 and vaccination with messenger RNA (mRNA) constructs encoding the viral spike protein typically generate effective immunity against COVID-19. However, the current pandemic has been fueled by the continual emergence of VOCs, such as Omicron (B.1.1.529). Recent data indicate that B.1.1.529 is more transmissible than previous VOCs^1^. This phenotype can be explained by key mutations in the receptor-binding domain, which confer enhanced affinity for the ACE2 receptor^2^. Another major concern is that B.1.1.529 harbors a large number of additional mutations in the spike protein that could feasibly subvert immune recognition (https://www.ecdc.europa.eu/sites/default/files/documents/threat-assessment-covid-19-emergence-sars-cov-2-variant-omicron-december-2021.pdf). In line with this possibility, emerging reports have shown that neutralizing antibodies elicited against the ancestral Wuhan reference strain, in the context of either infection or vaccination, are less able to combat B.1.1.529 (refs. ^2,3^). These observations likely align with the propensity of B.1.1.529 to cause breakthrough infections^4,5^.

Preliminary data suggest that breakthrough infections with B.1.1.529 are associated with a lower risk of hospitalization and/or severe illness compared with the Delta VOC (B.1.617.2) (refs. ^6,7^). One possible inference from these clinical observations is that additional immune mechanisms beyond antibody production attenuate the course of infection with B.1.1.529. Previous studies have demonstrated that robust CD4^+^ and CD8^+^ T cell responses are induced following SARS-CoV-2 infection or vaccination^8–14^. Several lines of evidence further suggest that CD4^+^ and CD8^+^ T cell responses can modulate disease severity in humans and suppress viral replication in animal models^15–18^. However, it has remained unclear to what extent ancestral SARS-CoV-2-specific CD4^+^ and CD8^+^ T cells cross-recognize B.1.1.529, especially given the unprecedented number of mutations in the spike protein, which likely shift the antigenic landscape more profoundly in relation to antecedent VOCs^19^.

**Extended Data Fig. 1:**
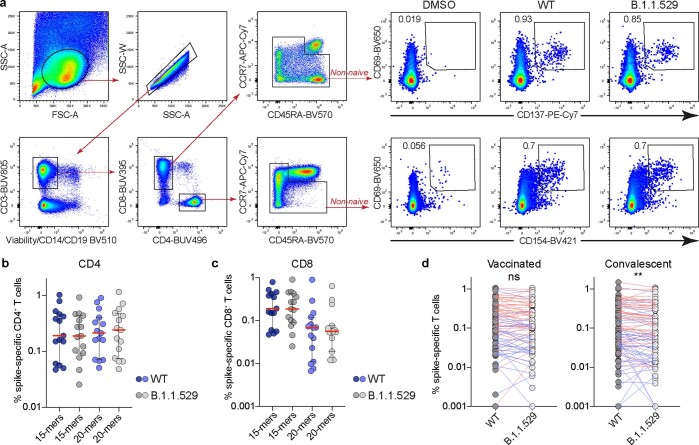
Peptide validation experiments. **a**, Representative flow cytometry plots showing the gating strategy used to assess spike-specific CD4^+^ and CD8^+^ T cell responses to peptide pools representing wild-type SARS-CoV-2 (WT) or B.1.1.529. **b**,**c**, Frequencies of spike-specific CD4^+^ (**b**) and CD8^+^ T cells (**c**) in BNT162b2-vaccinated individuals, comparing test 15mer peptide pools *versus* validation 20mer peptide pools representing wild-type SARS-CoV-2 (WT) or B.1.1.529. **d**, Pairwise analysis of spike-specific CD4^+^ (red lines) and CD8^+^ T cell responses (blue lines) in BNT162b2-vaccinated and convalescent individuals. Data in dot plots are shown as median ± IQR. Each dot represents one donor. **P = 0.007 (Two-sided Wilcoxon signed rank test). ns, not significant.

To address this question, we collected peripheral blood mononuclear cells (PBMCs) from vaccinated individuals 6 months after a second dose of the Pfizer/BioNTech mRNA BNT162b2 formulation (median age, 53 years; *n* = 23 females and 17 males), individuals in the convalescent phase 9 months after mild (median age, 54 years; *n* = 8 females and 18 males) or severe COVID-19 (median age, 58 years; *n* = 3 females and 19 males) and seronegative individuals (unclassified demographics, total *n* = 48) (Supplementary Table 1). Cells were stimulated in parallel with overlapping peptide pools spanning the entire spike protein sequences of the Wuhan reference strain (wild-type) or B.1.1.529. Activation-induced marker assays were used to quantify spike-specific CD4^+^ T cell responses via the upregulation of CD69 and CD40L (CD154) and spike-specific CD8^+^ T cell responses via the upregulation of CD69 and 4-1BB (CD137) (Extended Data Fig. 1a).

**Fig. 1:**
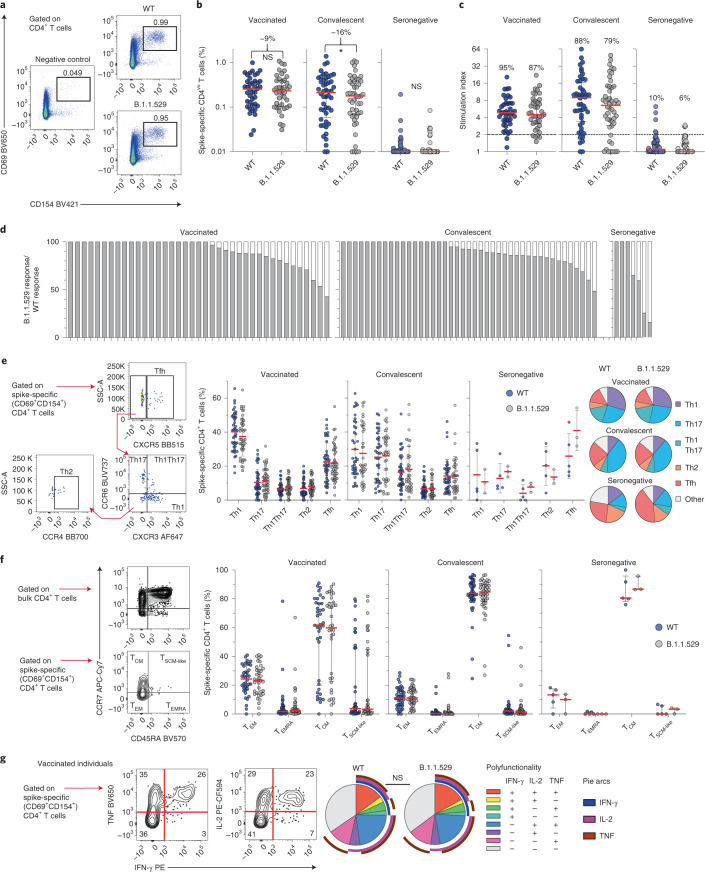
Cross-reactive CD4^+^ T cell responses against B.1.1.529. **a**, Representative flow cytometry plots showing spike-specific CD4^+^ T cell responses (CD69^+^CD154^+^) to peptide pools representing wild-type SARS-CoV-2 (WT) or B.1.1.529. **b**, Frequencies of all spike-specific CD4^+^ T cells in BNT162b2-vaccinated, convalescent and seronegative individuals. Numbers indicate median reduction in the frequency of detected responses. Comparisons used two-sided Wilcoxon signed rank tests. *P = 0.012. **c**, Stimulation indices calculated as fold change in frequency relative to the negative control. Numbers indicate the percentage of individuals with a detectable response. **d**, Cross-reactive responses depicted on an individual basis as percentage B.1.1.529/wild-type. **e**, Helper polarization of spike-specific CD4^+^ T cells with representative gating and dot plots showing the distribution of subsets across individuals with detectable responses. Pie charts show the mean frequency of each subset across all individuals in each group. **f**, Canonical memory differentiation profiles of spike-specific CD4^+^ T cells with representative gating and dot plots showing the distribution of subsets across individuals with detectable responses. **g**, Functional profiles of spike-specific CD4^+^ T cell responses in BNT162b2-vaccinated individuals with representative gating and pie charts showing the mean frequency for each combination. Polyfunctional responses were compared using a permutation test. Data in dot plots are shown as median ± interquartile range. Each dot represents one donor.

The overall magnitude of the SARS-CoV-2 spike-specific CD4^+^ T cell response against B.1.1.529 showed a median reduction of 9% in BNT162b2-vaccinated individuals and a median reduction of 16% in convalescent individuals relative to the wild-type response (Fig. 1a,b). The corresponding response frequencies, defined using a threshold stimulation index, were also slightly lower for B.1.1.529 (Fig. 1c). Pairwise comparisons further revealed maximum reductions in magnitude of 58% among BNT162b2-vaccinated individuals who were vaccinated, 56% among convalescent individuals and 75% among individuals who were seronegative for SARS-CoV-2 spike-specific CD4^+^ T cell responses against B.1.1.529 versus wild-type (Fig. 1d). These results were validated using independently synthesized peptide pools spanning each spike protein (Extended Data Fig. 1b).

To extend these findings, we investigated the phenotypic characteristics of SARS-CoV-2 spike-specific CD4^+^ T cells that cross-recognized B.1.1.529, with a particular focus on markers of T helper polarization (CCR4, CCR6, CXCR3 and CXCR5) and memory differentiation (CCR7 and CD45RA). No significant differences in T helper polarization were detected across intragroup comparisons of SARS-CoV-2 spike-specific CD4^+^ T cell responses against B.1.1.529 versus wild-type (Fig. 1e). Central memory T cells predominated among SARS-CoV-2 spike-specific CD4^+^ T cells in BNT162b2-vaccinated, convalescent and seronegative individuals, but again, no significant differences in subset composition were detected across intragroup comparisons of SARS-CoV-2 spike-specific CD4^+^ T cell responses against B.1.1.529 versus wild-type (Fig. 1f). We also assessed the functionality of SARS-CoV-2 spike-specific CD4^+^ T cells in BNT162b2-vaccinated individuals, measuring the intracellular expression of interferon-γ, tumor necrosis factor and interleukin-2 alongside CD69 and CD154. No significant differences in the ability of SARS-CoV-2 spike-specific CD4^+^ T cells to deploy multiple functions were apparent in response to stimulation with peptides representing B.1.1.529 versus wild-type (Fig. 1g).

**Fig. 2:**
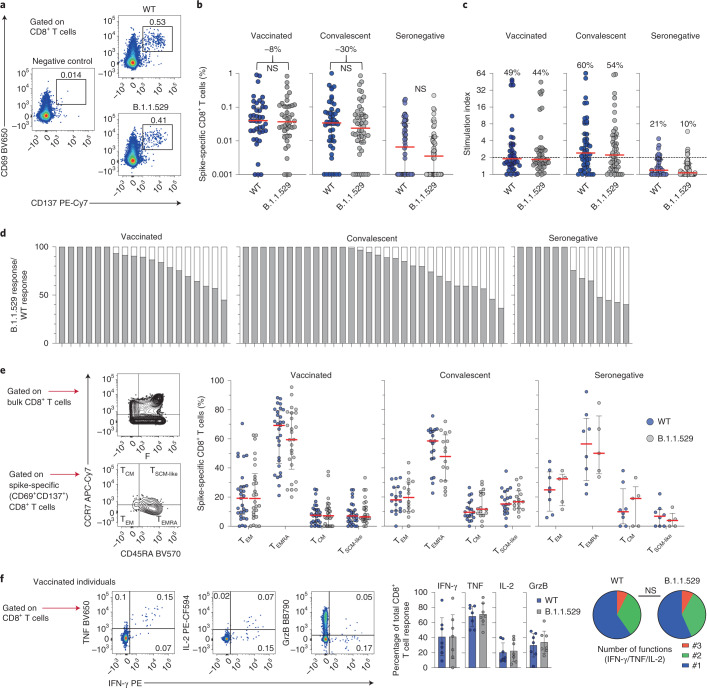
Cross-reactive CD8^+^ T cell responses against B.1.1.529. **a**, Representative flow cytometry plots showing spike-specific CD8^+^ T cell responses (CD69^+^CD137^+^) to peptide pools representing wild-type SARS-CoV-2 (WT) or B.1.1.529. **b**, Frequencies of all spike-specific CD8^+^ T cells in BNT162b2-vaccinated, convalescent and seronegative individuals. Numbers indicate median reduction in the frequency of detected responses. Comparisons used two-sided Wilcoxon signed rank tests. **c**, Stimulation indices calculated as fold change in frequency relative to the negative control. Numbers indicate the percentage of individuals with a detectable response. **d**, Cross-reactive responses depicted on an individual basis as percentage B.1.1.529/wild-type. **e**, Canonical memory differentiation profiles of spike-specific CD8^+^ T cells with representative gating and dot plots showing the distribution of subsets across individuals with detectable responses. **f**, Functional profiles of spike-specific CD8^+^ T cells in BNT162b2-vaccinated individuals with representative gating and pie charts showing the mean frequency for each combination. Polyfunctional responses were compared using a permutation test. Data in bar charts are shown as mean ± 95% confidence intervals, and data in dot plots are shown as median ± interquartile range. Each dot represents one donor. GrzB, granzyme B; NS, not significant.

The overall magnitude of the SARS-CoV-2 spike-specific CD8^+^ T cell response against B.1.1.529 showed a median reduction of 8% in BNT162b2-vaccinated individuals and a median reduction of 30% in convalescent individuals relative to the wild-type response (Fig. 2a,b). These differences were mirrored in the corresponding response frequencies, defined using a threshold stimulation index (Fig. 2c). Pairwise comparisons further revealed maximum reductions in magnitude of 55% among BNT162b2-vaccinated individuals, 63% among convalescent individuals and 60% among seronegative individuals for SARS-CoV-2 spike-specific CD8^+^ T cell responses against B.1.1.529 versus wild-type (Fig. 2d). These results were again validated using independently synthesized peptide pools spanning each spike protein (Extended Data Fig. 1c).

In further experiments, we investigated the phenotypic characteristics of SARS-CoV-2 spike-specific CD8^+^ T cells that cross-recognized B.1.1.529, focusing on classic markers of memory differentiation (CCR7 and CD45RA). Late effector memory T cells predominated among SARS-CoV-2 spike-specific CD8^+^ T cells in BNT162b2-vaccinated, convalescent and seronegative individuals, but no significant differences in subset composition were detected across intragroup comparisons of SARS-CoV-2 spike-specific CD8^+^ T cell responses against B.1.1.529 versus wild-type (Fig. 2e). We also assessed the functionality of SARS-CoV-2 spike-specific CD8^+^ T cells in BNT162b2-vaccinated individuals, measuring the intracellular expression of granzyme B, interferon-γ, tumor necrosis factor and interleukin-2 alongside CD69 and CD137. Akin to the corresponding analyses of SARS-CoV-2 spike-specific CD4^+^ T cells, no significant differences in the ability of SARS-CoV-2 spike-specific CD8^+^ T cells to deploy multiple functions were apparent in response to stimulation with peptides representing B.1.1.529 versus wild-type (Fig. 2f).

Finally, we merged the SARS-CoV-2 spike-specific CD4^+^ and CD8^+^ T cell data by group, aiming to evaluate cross-recognition en masse. The overall magnitude of the combined SARS-CoV-2 spike-specific CD4^+^ and CD8^+^ T cell response against B.1.1.529 was significantly lower in convalescent individuals, but not in BNT162b2-vaccinated individuals, relative to the wild-type response (Extended Data Fig. 1d). Although potentially reflecting differences in the chronology and/or context of antigen exposure, these results suggest that ancestral SARS-CoV-2 spike-specific CD4^+^ and CD8^+^ T cells elicited by natural infection provide comprehensive but relatively incomplete coverage against B.1.1.529.

The current global pandemic has been destabilized by the recent emergence of B.1.1.529, which continues to spread rapidly and supersede other VOCs. Our collective data indicate that SARS-CoV-2 spike-specific CD4^+^ and CD8^+^ T cells elicited by BNT162b2 vaccination or prior infection remain largely intact against B.1.1.529. Alongside intrinsic viral factors, such as altered tropism and decreased replication in the lower respiratory tract^20^, such heterologous immune reactivity may explain why severe disease appears to be relatively uncommon after infection with this particular VOC. Moreover, the degree of cross-reactivity varied to some extent among individuals, most likely as a consequence of genetically encoded differences in antigen presentation, which could further modulate clinical outcomes associated with B.1.1.529. It should be noted that we did not formally assess cytotoxic functions beyond the expression of granzyme B and that our evaluations were confined to peripheral blood samples, which do not necessarily reflect the entirety of the cellular immune response against SARS-CoV-2 (ref. ^21^). In addition, we found that SARS-CoV-2 spike-specific CD4^+^ and CD8^+^ T cells cross-recognized B.1.1.529 less comprehensively in convalescent versus BNT162b2-vaccinated individuals, suggesting that booster immunization may provide benefits that extend beyond the induction of broadly neutralizing antibodies to enhance natural protection against recurrent episodes of COVID-19 (ref. ^2^).

## Methods

### Samples

Healthy individual volunteers (*n* = 40) were sampled 6 months after a second dose of the BNT162b2 vaccine (Pfizer/BioNTech) as part of a clinical trial registered at EudraCT (2021-000175-37) (ref. ^22^). Two standard doses of the vaccine were administered with an interval of 21 days. The study was approved by the Swedish Medical Product Agency (ID 5.1-2021-5881). Additional samples (*n* = 15) were collected 3 months after the second dose for validation purposes (Extended Data Fig. 1). Convalescent individual volunteers were sampled 9 months after reverse-transcription polymerase chain reaction-verified infection with SARS-CoV-2 leading to mild (nonhospitalized, *n* = 26) or severe (hospitalized, *n* = 22) disease during the first wave of the pandemic in March–April 2020, before the emergence of the Alpha, Beta and Delta VOCs. None of these individuals had received a COVID-19 vaccine at the time of sample collection. Seronegative volunteer samples were acquired from healthy blood donors in late 2020. The absence of spike-specific antibodies was confirmed using the Anti-SARS-CoV-2 S Immunoassay (Roche). Cohort details are summarized in Supplementary Table 1. All participants provided written informed consent in accordance with the principles of the Declaration of Helsinki. Convalescent and seronegative cohorts were approved by the Regional Ethics Committee in Stockholm, Sweden. Population characteristics of each cohort were not considered and did not factor in for inclusion into this study. PBMCs were isolated via standard density gradient centrifugation and cryopreserved in fetal bovine serum (FBS) containing 10% dimethyl sulfoxide (DMSO).

### Peptides

Overlapping peptides were designed to span the entire spike protein sequence of SARS-CoV-2 corresponding to the ancestral Wuhan strain (wild-type) or B.1.1.529. Test peptides comprising 15mers overlapping by ten amino acids were synthesized as crude material for functional screens (TC Peptide Lab). Validation peptides comprising 20mers overlapping by ten amino acids were synthesized to an equivalent specification (Sigma-Aldrich). All peptides were reconstituted in DMSO, diluted to stock concentrations of 100 μg ml^−1^ in phosphate-buffered saline (PBS) and stored at −20 °C.

### Activation-induced marker assays

PBMCs were thawed quickly; resuspended in RPMI 1640 containing 10% FBS, 1% l-glutamine and 1% penicillin/streptomycin (complete medium) in the presence of DNase I (10 U ml^−1^, Sigma-Aldrich); and rested at 1 × 10^6^ cells per well in 96-well U-bottom plates (Corning) for 4 h at 37 °C. The medium was then supplemented with anti-CXCR5-BB515 and anti-CD40 (unconjugated), followed 15 min later by the relevant peptide pools (1 μg ml^−1^ per peptide). Negative control wells contained equivalent DMSO. After 12 h, cells were washed in PBS supplemented with 2% FBS and 2 mM EDTA (FACS buffer) and stained with anti-CCR4/CD194–BB700, anti-CCR6/CD196–BUV737, anti-CCR7–APC-Cy7 and anti-CXCR3-AF647 for 10 min at 37 °C. Additional surface stains were performed for 30 min at room temperature in the presence of Brilliant Stain Buffer Plus (BD Biosciences). Viable cells were identified by exclusion using a LIVE/DEAD Fixable Aqua Dead Cell Stain Kit (Thermo Fisher Scientific). Stained cells were washed in FACS buffer, fixed in PBS containing 1% paraformaldehyde (Biotium), and acquired using a FACSymphony A5 (BD Biosciences). The gating strategy is shown in Extended Data Fig. 1. All flow cytometry reagents are detailed in Supplementary Table 2.

### Intracellular cytokine staining

PBMCs were thawed quickly, resuspended in complete medium in the presence of DNase I (10 U ml^−1^, Sigma-Aldrich) and rested at 1 × 10^6^ cells per well in 96-well U-bottom plates (Corning) for 4 h at 37 °C. The medium was then supplemented with anti-CXCR5–BB515, followed 15 min later by the relevant peptide pools (1 μg ml^−1^ per peptide) and a further 1 h later by brefeldin A (1 μg ml^−1^, Sigma-Aldrich), monensin (0.7 μg ml^−1^, BD Biosciences) and anti-CD107a–BV785. Negative control wells contained equivalent DMSO. After 9 h, cells were washed in FACS buffer and stained with anti-CCR4/CD194–BB700, anti-CCR6/CD196–BUV737, anti-CCR7–APC-Cy7 and anti-CXCR3–BV750 for 10 min at 37 °C. Additional surface stains were performed for 30 min at room temperature in the presence of Brilliant Stain Buffer Plus (BD Biosciences). Viable cells were identified by exclusion using a LIVE/DEAD Fixable Aqua Dead Cell Stain Kit (Thermo Fisher Scientific). Cells were then washed in FACS buffer and fixed/permeabilized using a FoxP3 Transcription Factor Staining Buffer Set (Thermo Fisher Scientific). Intracellular stains were performed for 30 min at room temperature. Stained cells were washed in FACS buffer, fixed in PBS containing 1% paraformaldehyde (Biotium) and acquired using a FACSymphony A5 (BD Biosciences). All flow cytometry reagents are detailed in Supplementary Table 2.

### Data analysis and statistics

All samples from each cohort were randomly assigned and analyzed with wild-type and Omicron variant peptides in the same experiment. Flow cytometry data were analyzed using FlowJo version 10.8.0 (FlowJo). Stimulation indices were calculated as fold change in frequency relative to the negative control (equivalent DMSO). Positive responses were identified using a threshold stimulation index >2 to exclude background or nonspecific responses. Only memory populations were included for the analysis of spike-specific responses by the exclusion of the naive subset (CD45RA^+^CCR7^+^). Data exclusion criteria were established before all experiments. The investigators were not blinded to allocation during experiments and outcome assessment. Statistical analyses were performed using Prism version 9 (GraphPad). Significance between paired groups was assessed using two-sided Wilcoxon signed rank tests. Functional profiles were deconvoluted using Boolean gating in FlowJo version 10.8.0 (FlowJo) followed by downstream analyses in SPICE version 6.1 (https://niaid.github.io/spice/).

### Reporting Summary

Further information on research design is available in the Nature Research Reporting Summary linked to this article.

## Online content

Any methods, additional references, Nature Research reporting summaries, source data, extended data, supplementary information, acknowledgements, peer review information; details of author contributions and competing interests; and statements of data and code availability are available at 10.1038/s41591-022-01700-x.

## Supplementary information

Supplementary Information Supplementary Tables 1 and 2.

Reporting Summary

## Data Availability

All requests for raw and analyzed preclinical data and materials will be promptly reviewed by the corresponding author (M.B.) to determine if they are subject to intellectual property or confidentiality obligations. Any data and materials that can be shared will be released via a material transfer agreement (requested to M.B.). Personal data underlying this article cannot be shared publicly as they are sensitive. Enquiries regarding data availability should be directed to marcus.buggert@ki.se.
