# Supplementary material for: Ancestral SARS-CoV-2-specific T cells cross-recognize the Omicron variant
Source: Nat Med. 2022 Jan 14;28(3):472–6. doi: 10.1038/s41591-022-01700-x (PMC8938268; doi:10.1038/s41591-022-01700-x)
Supplement: Supplementary file 1 — Supplementary Tables 1 and 2. [file 41591_2022_1700_MOESM1_ESM.pdf]

nature portfolio

https://doi.org/10.1038/s41591-022-01700-x

Supplementary information

Ancestral SARS-CoV-2-specific T cells cross-recognize the Omicron variant

In the format provided by the authors and unedited

Supplementary Table 1. Cohort summary.

	**Vaccinated (n = 40)**	**Convalescent (mild, n = 26)**	**Convalescent (severe, n = 22)**	**Seronegative (n = 48)**
Age (years)				
Median	53	54	58	NA
Range	22–79	44–68	33–66	NA
Sex				
Male	17 (43%)	18 (69%)	19 (86%)	NA
Female	23 (58%)	8 (31%)	3 (14%)	NA
Required intensive care	0	0	14 (64%)	0
Spike-specific antibodies at recruitment*	ND	**	**	ND

*Measured using an anti-SARS-CoV-2 S Immunoassay. **Infections confirmed by RT-PCR for SARS-CoV-2. NA, not available; ND, not detected.

Supplementary Table 2. Flow cytometry reagents.

Antigen	Clone	Fluorophore	Company	Panel	Catalogue No.	Dilution
Viability	-	Fixable Aqua	Thermo Fisher	surface + intracellular	L34957	3:5000
CCR7	G043H7	APC-Cy7	BioLegend	surface + intracellular	353212	1:50
CD3	UCHT1	BUV805	BD Biosciences	surface + intracellular	612895	Surface 1:50<br>Intracellular 1:250
CD4	SK3	BUV496	BD Biosciences	surface + intracellular	612936	1:25
CD8	RPA-T8	BUV395	BD Biosciences	surface + intracellular	563795	1:250
CD14	M5E2	BV510	BioLegend	surface + intracellular	301842	1:100
CD19	HIB19	BV510	BioLegend	surface + intracellular	302242	1:100
CD45RA	HI100	BV570	BioLegend	surface + intracellular	304132	1:200
CD137	4B4-1	PE-Cy7	BioLegend	surface + intracellular	309818	Surface 1:25<br>Intracellular 1:100
CD154	24-31	BV421	BioLegend	surface + intracellular	310824	1:25
CD194	1G1	BB700	BD Biosciences	surface + intracellular	566475	1:50
CD196	11A9	BUV737	BD Biosciences	surface + intracellular	612780	1:500
CXCR5	RF8B2	BB515	BD Biosciences	surface + intracellular	564624	1:100
CD40	HB14	Unconjugated	Miltenyi Biotec	surface	130-094-133	1:200
CD69	FN50	BV650	BioLegend	surface	310934	1:50
CXCR3	G025H7	AF647	BioLegend	surface	353712	1:200
CD69	FN50	BUV563	BD Biosciences	intracellular	748764	1:200
CD107a	H4A3	BV785	BioLegend	Intracellular	328644	1:500
CXCR3	1C6	BV750	BD Biosciences	intracellular	746895	1:50
GrzB	GB11	BB790	BD Biosciences	intracellular	624296	1:500
IFN-γ	B27	PE	BioLegend	intracellular	506507	1:400
IL-2	MQ1-17H12	PE-Dazzle594	BioLegend	intracellular	500344	3:100
PD-1	EH12.2H7	BV711	BioLegend	intracellular	329928	1:25
TNF	MAb11	BV650	BD Biosciences	intracellular	563418	3:500
