# Supplementary material for: Ancestral SARS-CoV-2-specific T cells cross-recognize the Omicron variant
Source: Nat Med. 2022 Jan 14;28(3):472–6. doi: 10.1038/s41591-022-01700-x (PMC8938268; doi:10.1038/s41591-022-01700-x)
Supplement: Supplementary file 2 — Reporting Summary [file 41591_2022_1700_MOESM2_ESM.pdf]

nature portfolio

Corresponding author(s): Marcus Buggert

Last updated by author(s): Jan 13, 2022

Reporting Summary

Nature Portfolio wishes to improve the reproducibility of the work that we publish. This form provides structure for consistency and transparency in reporting. For further information on Nature Portfolio policies, see our Editorial Policies and the Editorial Policy Checklist.

Statistics

For all statistical analyses, confirm that the following items are present in the figure legend, table legend, main text, or Methods section.

n/a	Confirmed	
☐	☒	The exact sample size (*n*) for each experimental group/condition, given as a discrete number and unit of measurement
☐	☒	A statement on whether measurements were taken from distinct samples or whether the same sample was measured repeatedly
☐	☒	The statistical test(s) used AND whether they are one- or two-sided<br>*Only common tests should be described solely by name; describe more complex techniques in the Methods section.*
☒	☐	A description of all covariates tested
☒	☐	A description of any assumptions or corrections, such as tests of normality and adjustment for multiple comparisons
☐	☒	A full description of the statistical parameters including central tendency (e.g. means) or other basic estimates (e.g. regression coefficient) AND variation (e.g. standard deviation) or associated estimates of uncertainty (e.g. confidence intervals)
☐	☒	For null hypothesis testing, the test statistic (e.g. *F*, *t*, *r*) with confidence intervals, effect sizes, degrees of freedom and *P* value noted<br>*Give P values as exact values whenever suitable.*
☒	☐	For Bayesian analysis, information on the choice of priors and Markov chain Monte Carlo settings
☒	☐	For hierarchical and complex designs, identification of the appropriate level for tests and full reporting of outcomes
☒	☐	Estimates of effect sizes (e.g. Cohen's *d*, Pearson's *r*), indicating how they were calculated

Our web collection on statistics for biologists contains articles on many of the points above.

Software and code

Policy information about availability of computer code

Data collection	Flow cytometry data was collected using a BD FACSymphony A5 flow cytometer.
Data analysis	Flow cytometry data was analyzed using FlowJo (Version 10.8.0) and GraphPad Prism (Version 9.0.0).<br>Polyfunctional analysis was performed using SPICE (Version 6) (available at https://niaid.github.io/spice/).

For manuscripts utilizing custom algorithms or software that are central to the research but not yet described in published literature, software must be made available to editors and reviewers. We strongly encourage code deposition in a community repository (e.g. GitHub). See the Nature Portfolio guidelines for submitting code & software for further information.

Data

Policy information about availability of data

All manuscripts must include a data availability statement. This statement should provide the following information, where applicable:

- Accession codes, unique identifiers, or web links for publicly available datasets
- A description of any restrictions on data availability
- For clinical datasets or third party data, please ensure that the statement adheres to our policy

All requests for raw and analyzed preclinical data and materials will be promptly reviewed by the corresponding author (M.B.) to determine if they are subject to intellectual property or confidentiality obligations. Any data and materials that can be shared will be released via a material transfer agreement (requested to M.B.). Personal data underlying this article cannot be shared publicly as they are sensitive. Enquiries regarding data availability should be directed to marcus.buggert@ki.se.

Field-specific reporting

Please select the one below that is the best fit for your research. If you are not sure, read the appropriate sections before making your selection.

☒ Life sciences ☐ Behavioural & social sciences ☐ Ecological, evolutionary & environmental sciences

For a reference copy of the document with all sections, see nature.com/documents/nr-reporting-summary-flat.pdf

Life sciences study design

All studies must disclose on these points even when the disclosure is negative.

Sample size	With a sample size of n = 20 per group, the probability is 80% that the study will detect a relationship between the independent and the dependent variables at a two-sided 0.05 significance level, if the true change in the dependent variables is 0.663 standard deviations per one standard deviation change in the independent variable. Based on previous experience (Sekine et al, 2020, Cell etc), it should therefore be possible to detect group differences.<br>The total number of individuals from each cohort were selected to match as closely as possible and be approximately twice as high as our power analysis. Vaccinated n = 40. Convalescent n = 48. Seronegative n = 48.
Data exclusions	Individuals with a stimulation index less than 2 were excluded from downstream phenotypic and functional analyses to minimise analysis of background or non-specific responses. Only memory populations were included for the analysis of spike-specific responses by the exclusion of the naive subset (CD45RA+CCR7+). Data exclusion criteria were established before all experiments, similar to before Niessl et al, 2020, Science Immunol etc).
Replication	Given limited sample availability, replication was not performed.
Randomization	Individuals were randomly analyzed. However, WT and Omicron peptides were supplemented in the same experiments to avoid intra-individual experimental differences of T cell responses against the different viral variants.
Blinding	Investigators were not blinded. The data generation for all samples within the same cohort were run in parallel in single experiments.

Reporting for specific materials, systems and methods

We require information from authors about some types of materials, experimental systems and methods used in many studies. Here, indicate whether each material, system or method listed is relevant to your study. If you are not sure if a list item applies to your research, read the appropriate section before selecting a response.

Materials & experimental systems

n/a	Involved in the study	
☐	☒	Antibodies
☒	☐	Eukaryotic cell lines
☒	☐	Palaeontology and archaeology
☒	☐	Animals and other organisms
☐	☒	Human research participants
☒	☐	Clinical data
☒	☐	Dual use research of concern

Methods

n/a	Involved in the study	
☒	☐	ChIP-seq
☐	☒	Flow cytometry
☒	☐	MRI-based neuroimaging

Antibodies

Antibodies used

AIM assay:
Fixable Aqua Viability dye Thermo Fisher Cat#L34957 Dilution 3:5000
BUV805 CD3 BD Biosciences Clone UCHT1 Cat#612895 Dilution 1:50
BUV496 CD4 BD Biosciences Clone SK3 Cat#612936 Dilution 1:25
BUV395 CD8 BD Biosciences Clone RPA-T8 Cat#563795 Dilution 1:250
BV510 CD14 BioLegend Clone M5E2 Cat#301842 Dilution 1:100
BV510 CD19 BioLegend Clone HIB19 Cat#302242 Dilution 1:100
BV570 CD45RA BioLegend Clone HI100 Cat#304132 Dilution 1:200
APC-Cy7 CCR7 BioLegend Clone G043H7 Cat#353212 Dilution 1:50
PE-Cy7 CD137 BioLegend Clone 4B4-1 Cat#309818 Dilution 1:25
BV421 CD154 BioLegend Clone 24-31 Cat#310824 Dilution 1:25
BB700 CD194 BD Biosciences Clone 1G1 Cat#566475 Dilution 1:50
BUV737 CD196 BD Biosciences Clone 11A9 Cat#612780 Dilution 1:500
BB515 CXCR5 BD Biosciences Clone RF8B2 Cat#564624 Dilution 1:100
Unconjugated CD40 Miltenyi Biotec Clone HB14 Cat#130-094-133 Dilution 1:200
BV650 CD69 BioLegend Clone FN50 Cat#310934 Dilution 1:50

AF647 CXCR3 BioLegend Clone G025H7 Cat#353712 Dilution 1:200
Intracellular staining:
Fixable Aqua Viability dye Thermo Fisher Cat#L34957 Dilution 3:5000
BUV805 CD3 BD Biosciences Clone UCHT1 Cat#612895 Dilution 1:250
BUV496 CD4 BD Biosciences Clone SK3 Cat#612936 Dilution 1:25
BUV395 CD8 BD Biosciences Clone RPA-T8 Cat#563795 Dilution 1:250
BV510 CD14 BioLegend Clone M5E2 Cat#301842 Dilution 1:100
BV510 CD19 BioLegend Clone HIB19 Cat#302242 Dilution 1:100
BV570 CD45RA BioLegend Clone HI100 Cat#304132 Dilution 1:200
APC-Cy7 CCR7 BioLegend Clone G043H7 Cat#353212 Dilution 1:50
PE-Cy7 CD137 BioLegend Clone 4B4-1 Cat#309818 Dilution 1:100
BV421 CD154 BioLegend Clone 24-31 Cat#310824 Dilution 1:25
BB700 CD194 BD Biosciences Clone 1G1 Cat#566475 Dilution 1:50
BUV737 CD196 BD Biosciences Clone 11A9 Cat#612780 Dilution 1:500
BB515 CXCR5 BD Biosciences Clone RF8B2 Cat#564624 Dilution 1:100
BUV563 CD69 BD Biosciences Clone FN50 Cat#748764 Dilution 1:200
BV785 CD107a BioLegend Clone H4A3 Cat#328644 Dilution 1:500
BV750 CXCR3 BD Biosciences Clone 1C6 Cat#746894 Dilution 1:50
BB790 Granzyme B BD Biosciences Clone GB11 Cat#624296 Dilution 1:500
PE IFN-γ BioLegend Clone B27 Cat#506507 Dilution 1:400
PE-Dazzle594 IL-2 BioLegend Clone MQ1-17H12 Cat#500344 Dilution 3:100
BV711 PD1 BioLegend Clone EH12.2H7 Cat#329928 Dilution 1:25
BV650 TNF BD Biosciences Clone MAb11 Cat#563418 Dilution 3:500

Validation

All antibodies are validated by their respective manufacturers and are quality control tested by surface or intracellular immunofluorescent staining with flow cytometric analysis. For more information on the antibodies used, please visit bioledend.com, bdbiosciences.com, thermofisher.com and miltenyibiotec.com.

Human research participants

Policy information about studies involving human research participants

Population characteristics

Among the 40 vaccinated individuals, all were adults with an age range of 22-79 (median age of 53), with females comprising 58%. All 48 convalescent patients confirmed by positive RT-PCR results for SARS-CoV-2 fell within the age range of 44-68 years (median age of 56), with 23% of them being females. Personal information of all 48 individuals with seronegative for SARS-COVID-2 is not available. Population characteristics of each cohort were not considered and did not factor in for inclusion into this study.

Recruitment

The vaccinated healthy individuals were recruited between Feb-March 2021 at Karolinska University Hospital, Sweden in a clinical trial (EudraCT no. 2021-000175-37). Most of the healthy individuals were family members of the study participants with immunocompromised disorders (not included in the present study), recruited to the trial for COVID-19 vaccination. Inclusion criteria for healthy individuals were individual ≥ 18 years, no known immunosuppressive disease or treatment with significant co-morbidity according to the investigator's judgement. The exclusion criteria were previous or ongoing COVID-19, presence of coagulation disease, planned to receive other vaccine within 14 days prior to the first dose of the study vaccine or receive other vaccine from the time of the first study vaccine dose until 14 days after the second dose of study vaccine, pregnancy or breast-feeding, hypersensitivity to any of the active substance in the vaccine, cannot comprehend the information given to study participants for consent, or individuals of any other reasons judged by the investigator to be not suitable for inclusion in the study. One potential selection bias could be the participants willingness for COVID-19 vaccination and serial venipuncture performed in this study, but it is unlikely that this could have impacted any of the results in this study significantly.

The convalescent individuals have been tested positive for SARS-CoV-2 infection during March-April 2020 at Karolinska University Hospital. Patients with severe COVID-19 have been hospitalized, while those with mild COVID-19 have been followed-up at the outpatient clinic of the department of Infectious Diseases, Karolinska University Hospital. The patients were recruited while in the convalescence phase, with blood samples in this study collected at 9 months after the verified SARS-CoV-2 infection. One potential selection bias could be the participants willingness for serial venipuncture or other features associated with individuals who declined enrollment into our study, but it is unlikely that this could have impacted any of the results significantly.

Seronegative samples were acquired from healthy blood donors in late 2020.

All study participants provided written informed consent. All study participants were volunteers.

Ethics oversight

The vaccinated cohort was approved by the Swedish Medical Product Agency (ID 5.1-2021-5881) and the Swedish Ethical Review Authority (ID 2021-00451). Other cohorts were approved by the Regional Ethics Committee in Stockholm, Sweden.

Note that full information on the approval of the study protocol must also be provided in the manuscript.

Flow Cytometry

Plots

Confirm that:

☒ The axis labels state the marker and fluorochrome used (e.g. CD4-FITC).

☒ The axis scales are clearly visible. Include numbers along axes only for bottom left plot of group (a 'group' is an analysis of identical markers).

☒ All plots are contour plots with outliers or pseudocolor plots.

☒ A numerical value for number of cells or percentage (with statistics) is provided.

Methodology

Sample preparation	Cryopreserved PBMC
Instrument	BD FACSymphony A5
Software	FlowJo version 10.8.0
Cell population abundance	No sorting was performed.
Gating strategy	Gating strategies are shown throughout the figures. Briefly, lymphocytes were gated by standard FSC/SSC gating followed by singlet discrimination. Viability staining was used to gate live cells. T cells were gated by CD3 expression and no expression of the lineage markers CD19 and CD14. CD4 and CD8 T cells were identified by CD4 and CD8 staining. Naive cells with CD45RA and CCR7 high expression were gated out. Within the remaining population, spike-specific CD4 and CD8 responses were identified by coexpression of CD69/CD154 or CD69/CD137 respectively.

☒ Tick this box to confirm that a figure exemplifying the gating strategy is provided in the Supplementary Information.
